# The fear of missing out and social media addiction: A cross-sectional and quasi-experimental approach

**DOI:** 10.1016/j.heliyon.2025.e41958

**Published:** 2025-01-15

**Authors:** Mohammed Hasan Ali Al-Abyadh

**Affiliations:** a College of Education in Al-Kharj, Prince Sattam Bin Abdulaziz University, Al-Kharj, 11942, Saudi Arabia; b College of Education, Thamar University, Dhamar, Yemen; c Al-Mutaqadima Schools' Company, Saudi Arabia

**Keywords:** The fear of missing out, Social media addiction, Prince Sattam Bin Abdulaziz University students

## Abstract

This research aimed to explore the connection between Fear of Missing Out (FoMO) and Social Media Addiction (SMA) and assess the efficacy of guidance and counseling programs in mitigating FoMO and SMA among Saudi students. Four hundred and seventy students from Prince Sattam Bin Abdulaziz University participated, completing the Fear of Missing Out Scale and the Bergen Social Media Addiction Scale. The study employed both a cross-sectional approach to examine associations among variables and a quasi-experimental method to gauge the impact of the Guidance and Counseling Program on reducing FoMO and SMA. Findings indicated a positive correlation between fear of missing out and social media addiction. Subsequent analysis of the experimental study demonstrated statistically significant differences in students' mean scores for FoMO and SMA before and after intervention. The experimental group exhibited significantly lower scores at the post-test compared to pretest scores. Conversely, there were no statistically significant differences in scores for the control groups before and after the intervention. In conclusion, guidance and counseling programs were found to be effective in diminishing the fear of missing out and social media addiction among students.

## 1 Introduction

The phenomenon of Fear of Missing Out (FoMO) has garnered significant attention in recent years, particularly due to its strong association with the rise of social media usage. FoMO is described as the anxiety or fear that one is missing out on rewarding experiences, important information, or opportunities, leading individuals to remain constantly connected to their social networks [1,2]. Rooted in the fear of remorse and social exclusion, FoMO drives people to compulsively engage with online platforms to avoid feelings of deprivation or social isolation [3,4]. Psychologists have linked this behavior to a broader fear of ostracism, as individuals continually compare their lives to others, often wondering whether they are missing out on better experiences or opportunities [5,6].

A recent study reported that 56 % of social media users experience FoMO, particularly when they are away from their accounts, leading to persistent feelings of anxiety and the urge to reconnect [7]. This connection between FoMO and social media addiction (SMA) has been well-documented in the literature, with studies like Zhu and Xiong [8] demonstrating that heightened FoMO correlates with increased SMA among students.

Despite this growing body of research, the present study aims to contribute to the existing literature by offering a more focused examination of how targeted interventions, such as guidance and counseling programs, can mitigate the impact of FoMO and SMA, particularly among Saudi students. Grounded in social comparison theory and self-determination theory, our research explores how unmet psychological and social needs drive individuals’ susceptibility to FoMO and SMA. By positioning our study within this theoretical framework and comparing our findings with existing research, we aim to offer novel insights into the effectiveness of interventions in reducing FoMO and SMA in educational settings.

### 1.1 The fear of missing out (FoMO)

In today's world, the notion that you might be missing out on something enjoyable is not new. Though there is evidence of fear of missing out (FoMO) in ancient literature, the concept has likely existed for centuries. Research on FoMO, however, dates back only a few decades, starting with a 1996 study by marketing expert Dr. Dan Herman, who first proposed the term “fear of missing out” [9]. Therefore, fear of missing out (FoMO) is considered the intense fear and anxiety of missing out on things or situations, such as social events, gatherings, and word of mouth [10,11]. So that the person feels that there is a gap between him and the latest events when he is unable to participate in them, or he feels that others are enjoying the time better than him. Fear of missing out (FoMO) is a common phenomenon that compels individuals to constantly stay connected, fearing that they might miss out on events or experiences in which they are not actively involved [12]. This condition is prevalent among the majority of social media users.

There is no specific reason for a person to suffer from FoMO, but some factors may play an important role in the matter, including the human instinct to communicate or stay in groups, as when a person feels that he has lost this ability to communicate, he has various fears. As well as the widespread use of the Internet and constant access to social media, this has led to comparing a person to others and scrutinizing what he lacks [13,14].

Researchers argued that younger people are considerably more at risk of FoMO [[15], [16], [17], [18]]. The prevalence of Fear of Missing Out (FoMO) is attributed to increased online activity and a heightened desire for social approval and a sense of belonging. It is important to note that FoMO is not limited to young individuals, as any frequent user of social media is more susceptible to experiencing FoMO compared to those who use social media sparingly [5,[19], [20], [21]].

FoMO can have distinct symptoms, but it is not currently a diagnosed ailment [[22], [23], [24]]. These symptoms include, among others: compulsion to constantly monitor social media to know what others are doing; having bad emotions while contrasting one's life with what other people appear to be doing on social media. In addition, overscheduling, or attempting to be everywhere at once [25,26].

### 1.2 The fear of missing out and social media addiction

The emergence of social media has significantly heightened the recognition and research surrounding Fear of Missing Out (FoMO). The prevalence of the FoMO phenomenon has been accelerated through various channels within the realm of social media. It creates an environment where you are contrasting the high points of other people's lives with your everyday existence. Social media addiction is defined as the compulsive use of social media platforms, which results in severe damage to the performance of individuals in various areas of life for a long period. The relationship between fear of missing out and addiction to social media is reciprocal, as one is a cause and effect of the other, as addiction to social media leads to fear of missing out, especially among teenagers or young people, and the fear of missing out or missing an important event leads a person to addiction to social media to avoid being interrupted from events [27].

A recent study investigating the relationship between high school-aged teens' social media addiction and FOMO discovered that there is an association between the two [28]. Another study conducted by Varchetta, Fraschetti, Mari, and Giannini [29] investigated the use of social networks among 306 Italian university students. The study found that there is a positive relationship between FoMO, online vulnerability, and social media addiction. In addition, results showed that FoMO is the best predictor of social media addiction.

Another research study examined how positive nonrecognition related to social media usage acts as a mediator between problematic social media use and fear of missing out (FoMO). The study involved 579 college students and considered factors such as the fear of negative evaluation and the perception of low self-presentational skills. The study found that both Fear of Missing Out (FoMO) and self-presentational skills were found to have direct and indirect associations with problematic social media use. These connections were mediated by positive meta-cognition. The results of this research revealed the significant effect of FoMO on problematic usage of social media [30].

There are several reasons why using social media might lead to increased symptoms of anxiety and melancholy, negative body image, trouble sleeping, and cyberbullying, but one of the strongest ones is increased social comparison. As per the social comparison hypothesis, people, while lacking objective knowledge, have a natural desire to compare themselves to others constantly in an effort to build an accurate self-scrutiny. Furthermore, social media platforms create new avenues for social comparison by providing users with a wealth of publicly available information about others.

Crusius, Corcoran, and Mussweiler [31] claim that a major factor influencing how people perceive, interpret, and act in social situations is social comparisons, or comparisons between oneself and others. According to the notion, people evaluate their social and personal worth by analyzing how they relate to and differ from other people. It also clarifies how people assess their own performance by contrasting their deeds, accomplishments, and viewpoints with those of other members of the community. Social media platforms offer a perfect setting for social comparison because they make it simple for users to showcase their accomplishments and contrast them with those of others. Feelings of inadequacy, insecurity, and FOMO may result from this.

However, self-determination theory is also predicated on the ways in which people interact with and rely on their social environments through the use of their motivation and personalities [32]. According to the notion, people have basic psychological requirements for relatedness, competence, and autonomy. The satisfaction of these requirements may be impacted by social media use, since excessive use can result in a decrease in feelings of competence, autonomy, and social connectivity.

Our research, which is based on the social comparison and self-determination theories, examines how people become more vulnerable to FoMO and SMA as a result of unfulfilled psychological and social demands. We hope to provide fresh perspectives on the efficacy of interventions in lowering FoMO and SMA in educational settings by situating our study within this theoretical framework and contrasting our results with other studies.

### 1.3 The current study

The Fear of Missing Out (FoMO) and social media addiction present not only individual challenges but also substantial societal concerns. Tackling these issues necessitates a holistic approach that integrates awareness, education, and support to foster healthier and more mindful social media usage. In Saudi Arabia, dependency on social networking sites among the youth has emerged as a pressing public health issue, as evidenced by a study conducted by Alfaya et al. [33], which found a 55.2 % prevalence of social media addiction among Saudi students in a selected university.

Furthermore, existing research indicates that individuals with lower life satisfaction are more prone to increased dependency on social media, and a heightened fear of missing out is a predictive factor for increased social media addiction [34].

Beyond individual consequences, several studies have demonstrated a negative correlation between social media addiction among Saudi students and academic performance [35]. Moreover, social media addiction has been positively associated with negative lifestyle, poor academic achievement [36], and maladjustment to college [37].

In light of these findings, our study aims to delve deeper into the intricate connection between social media addiction (SMA) and fear of missing out (FoMO) among Saudi students. Additionally, we seek to assess the effectiveness of guidance and counseling programs in alleviating the fear of missing out and mitigating social media addiction within this demographic. Through comprehensive research and targeted interventions, we aspire to contribute to the development of strategies that promote healthier digital habits and enhance the overall well-being of Saudi students. So, our hypotheses will be as follows.

H1 Fear of Missing Out (FoMO) positively correlates with Social Media Addiction (SMA) among Saudi students.

H2 A guidance and counseling program will decrease the feelings of Fear of Missing Out (FoMO) and Social Media Addiction (SMA) among Saudi students.

## 2 Method

The present study employs a mixed-method design consisting of two parts: a cross-sectional survey and a quasi-experimental intervention. The cross-sectional component aims to examine the relationship between Fear of Missing Out (FoMO), Social Media Addiction (SMA), and relevant demographic variables among Saudi students. While the quasi-experimental component evaluates the effectiveness of a guidance and counseling program in reducing levels of FoMO and SMA.

### 2.1 Participants and procedures

First, a total of 470 Prince Sattam Bin Abdulaziz University students voluntarily participated in this study. The age of the sample ranged from 18 to 24 years. All of them have at least one or more platforms of social media (Snapchat, Facebook, Instagram, Ticktock, YouTube, and Twitter), as shown in Table (1). The questionnaires were individually administered and took 20–25 min.

**Table 1:** Demographic characteristics of the principal study sample.

Variable	Frequency	Percent%
**Gender**		
Male	413	87.9 %
Female	57	12.1 %
**Level of study**		
1st	215	45.7 %
2nd	16	3.4 %
3rd	124	26.4 %
4th	54	11.5 %
5th	25	5.3 %
6th	13	2.8 %
7th	23	4.9 %
**Platform**		
One platform	69	14.7 %
Two platforms	68	14.5 %
Three platforms	66	14 %
Four platforms	54	11.5 %
Five platforms	50	10.6 %
All platforms	163	34.7 %

Second, 20 students from the principal sample who showed high levels of FoMO and SMA were recruited for the experimental work. They were divided into an experimental group (n = 10) and a control group (n = 10), as shown in Table (2). The experimental group received 12 sessions containing training, lectures, and instructions while the control group didn't receive any intervention. Finally, both experimental and control groups filled out the questionnaires again.

**Table 2:** Demographic characteristics of the experimental study.

Variable	Experimental group	Control group
**Gender**		
Male	5	5
Female	5	5
**Age**		
18 years old	1	2
19 years old	2	1
20 years old	1	2
21 years old	3	2
22 years old	2	2
24 years old	1	1

### 2.2 Tools

- ***Fear of Missing Out***: The Fear of Missing Out Scale of Przybylski, Murayama, DeHann, & Gladwell [2] was used. The Arabic version has demonstrated reliability and validity in Arabic contexts [38]. The Fear of Missing Out Scale consists of 10 items, with respondents rating each item on a 5-point Likert-type scale ranging from 1 (Not at all true of me) to 5 (Extremely true of me). The scale demonstrated good internal consistency, as indicated by a Cronbach's alpha coefficient of .78.
- ***Social Media Addiction***: The researchers utilized the Bergen Social Media Addiction Scale in this study. The Arabic version of this scale has been proven to be reliable and valid in Arabic contexts [39]. The scale consists of six items, and participants rated each item on a 5-point Likert-type scale ranging from 1 (very rarely) to 5 (very often). The Cronbach's alpha coefficient for the scale was calculated to be .87.
- ***The program***: The guidance and counseling program was developed by the author and underwent a thorough review process by five psychology professors, whose valuable input led to modifications. Before its full implementation, the program was pilot-tested on a smaller group of students to assess its effectiveness and make necessary adjustments based on feedback. The pilot test involved evaluating the program's content, delivery methods, and outcomes, ensuring its relevance and appropriateness for the target population. Additionally, the program's structure was validated to ensure it meets the intended objectives. The primary objective of this program is to reduce the fear of missing out (FoMO) and social media addiction among Saudi students. After making the necessary refinements, the program was administered to a substantial number of students in the experimental group through group counseling sessions. It incorporates techniques such as counseling, modeling, feedback, open dialogue, interactive discussions, informative lectures, and assigned homework. These sessions took place in a designated hall within the College of Education.

### 2.3 Data analysis

The study employed Pearson's correlation coefficients to analyze the relationships among the variables under investigation. Additionally, the Mann-Whitney *U* test and Wilcoxon signed rank test were employed to assess differences between the groups.

## 3 Results

Before beginning the analysis, the entire data set was evaluated for accuracy and normality. All scales in the primary sample showed acceptable values, indicating that the distribution of scores is normal and suitable for further data analysis, which will be as follows.

### 3.1 Correlations among the study variables

The results (Table 3) revealed that Fear of Missing Out (FoMO) was significantly correlated with Social Media Addiction (SMA). The p-value (.01) confirmed the statistical significance of the correlation.

**Table 3:** Correlation among the study variables (N = 470).

Variables	FoMO	SMA	*p*
**FoMO**	1		
**SMA**	.540∗∗	1	.01
Mean	3.61	3.53	
S D	.896	.538	

Note: ∗∗: p < 0.01. FoMO: Fear of Missing Out, SMA: Social Media Addiction.

### 3.2 The differences between groups

1. The differences between the groups before the intervention.

The findings from Table 4 indicated that there were no significant variations in the scores of Fear of Missing Out (FoMO) and Social Media Addiction (SMA) between the control and experimental groups before the intervention.

2. The differences between the groups after the intervention.

**Table 4:** Mann-Whitney *U* Test for pretest-experimental and control groups (N = 20).

Variable	Group	N	Mean Rank	Sum of Ranks	Mann-Whitney U	z	*p*
**FoMO**	*Experimental before*	10	10.90	109.00	46.000	−.304	.796
	*Control before*	10	10.10	101.00			
**SMA**	*Experimental before*	10	8.00	130.00	25.000	−1.903	.063
	*Control before*	10	13.00	80.00			

Note: ∗∗: p < 0.01. FoMO: Fear of Missing Out, SMA: Social Media Addiction.

**Figure (1):**
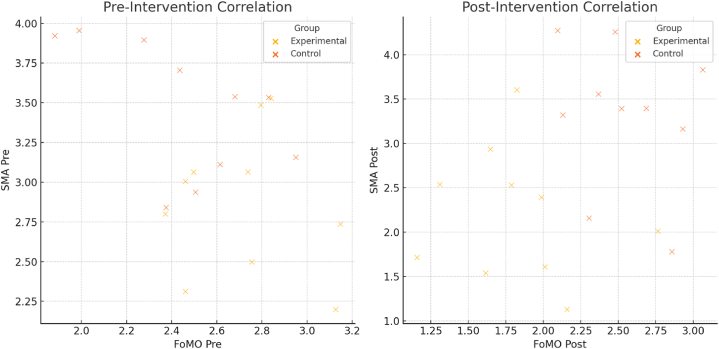
Correlation graph s of FOM and SMA pre- and post-intervention.

After the intervention, the results from Table 5 indicated significant statistical differences in the scores of Fear of Missing Out (FoMO) and Social Media Addiction (SMA) between both the control and experimental groups. For more details, Fig. 1 shows the correlation graphs of FOM and SMA pre- and post-intervention.

3. The differences between experimental groups

**Table 5:** Mann-Whitney *U* Test for post-experimental and control groups (N = 20).

Variable	Group	N	Mean Rank	Sum of Ranks	Mann-Whitney U	z	*p*
**FoMO**	*Experimental after*	10	8.10	81.00	26.000	−1.826	.000
	*Control after*	10	12.90	129.00			
**SMA**	*Experimental after*	10	6.25	62.50	7.500	−3.298	.032
	*Control after*	10	14.75	147.50			

Note: ∗∗: p < 0.01. FoMO: Fear of Missing Out, SMA: Social Media Addiction.

**Figure (2):**
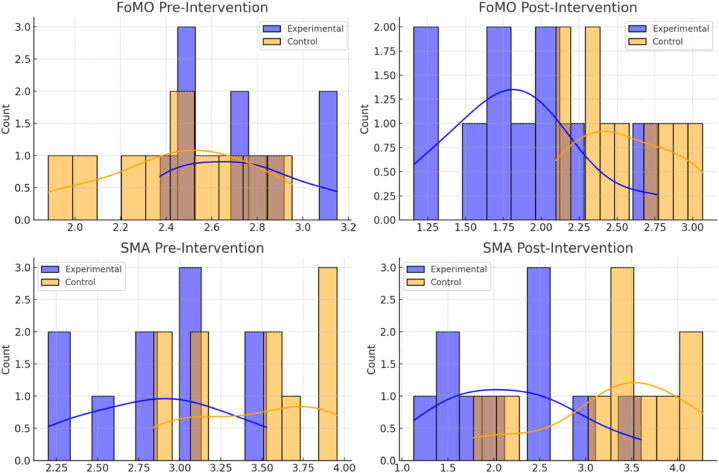
Histograms of FOM and SMA pre- and post-intervention.

The findings from Table 6 indicated significant statistical differences in the scores of Fear of Missing Out (FoMO) and Social Media Addiction (SMA) between the experimental groups before and after the intervention. For more details, Fig. 2 shows the histograms of FOM and SMA pre- and post-intervention.

**Table 6:** Wilcoxon Signed Rank test for pre-test and post-experimental groups.

	Experimental before		Experimental after		Z-test		
					N	Z	p
**FoMO**	*M*	*SD*	*M*	*SD*	10	−2.550	.011
	2.55	.379	1.95	.556			
**SMA**	*M*	*SD*	*M*	*SD*	10	−2.296	.022
	3.34	.596	2.40	.649			

Note: ∗∗: p < 0.01. FoMO: Fear of Missing Out, SMA: Social Media Addiction.

## 4 Discussion

The primary objective of this study was to investigate the association between Fear of Missing Out (FoMO) and Social Media Addiction (SMA) among Saudi students. The findings indicated a positive correlation between FoMO and SMA, suggesting that students with higher levels of FoMO are more prone to engaging in addictive behaviors associated with social media use. This result aligns with existing studies such as Kamaruddin, and Haris [[27], [28], [29]], which suggest that the constant connectivity provided by social media platforms may intensify feelings of anxiety and fear of exclusion and missing something, consequently fueling addictive patterns of usage. This study further contributes to the global understanding of this relationship by providing data specific to the Saudi context, a population that has been underrepresented in this field of research.

The second objective of this study was to evaluate the effectiveness of the Guidance and Counseling Program in lowering Fear of Missing Out (FoMO) and Social Media Addiction (SMA). The findings demonstrated statistically significant variations in the average scores of students who participated in the intervention, indicating a positive impact of counseling programs on both FoMO and SMA.These results are a significant contribution to the literature, as they highlight the potential of culturally tailored counseling interventions in addressing digital well-being issues specific to Saudi students. The program's impact is particularly noteworthy given the unique social and cultural dynamics of Saudi Arabia, which may influence students' interactions with social media.

Before the intervention, both the experimental and control groups were similar in terms of FoMO and SMA, indicating a good baseline. After the intervention, the experimental group showed significantly lower scores in both FoMO and SMA compared to the control group, highlighting the effectiveness of the intervention. Within the experimental group, there were significant reductions in FoMO and SMA scores post-intervention, further demonstrating the intervention's impact.

These findings support the conclusion that the intervention was effective in reducing both FoMO and SMA among participants in the experimental group.The experimental group exhibited scores on the post-test significantly lower than those on the pretest, emphasizing the potential of counseling interventions in addressing these issues. This result partially aligns with the findings of studies such as [40,41]; these studies found that the intervention and cognitive-behavioral therapy successfully alleviated symptoms associated with internet use disorder and Social Media Addiction.‏

Although the study makes valuable contributions, it is essential to acknowledge its limitations. The reliance on self-report measures may introduce response bias, and the generalization of the findings may be limited to the specific demographic of Prince Sattam Bin Abdulaziz University students. Future research could employ diverse methodologies, such as longitudinal studies, to explore the long-term effects of counseling interventions. Additionally, investigating the role of cultural factors in shaping FoMO and SMA within the Saudi context could provide further insights.

### 4.1 Implications

This study carries significant implications for educational institutions, especially given the rising prevalence of social media use among students in Saudi Arabia. The favorable outcomes observed in the Guidance and Counseling Program underscore the critical need for embedding mental health support services within academic institutions. One of the key contributions of this research is its demonstration of the efficacy of culturally sensitive counseling interventions in addressing the psychological impact of social media use. These results suggest that institutions should consider proactive measures to manage the psychological toll of social media on students, ultimately enhancing both their well-being and academic performance.

The research findings also offer valuable insights into the specific challenges faced by Saudi students in managing FoMO and SMA. By demonstrating the effectiveness of a tailored intervention program, this study makes a unique contribution to the growing field of cross-cultural research on digital well-being. It highlights the importance of customizing guidance and counseling programs to align with culturally relevant aspects of social media use, ensuring their effectiveness and resonance with the target population.

Moreover, the quasi-experimental approach adopted in this study serves as a model for future research and interventions. Replicating this study in diverse cultural or educational settings could further validate the efficacy of such programs and contribute to global efforts in promoting digital well-being. Beyond its immediate scope, this research makes a notable contribution to the broader discourse on individual and societal well-being in the digital age. By specifically addressing FoMO and SMA, it aligns with global initiatives to cultivate healthier digital habits and improve mental health outcomes, especially among student populations.

## 5 Conclusion

The present study investigated the relationship between the Fear of Missing Out (FoMO) and Social Media Addiction (SMA) among Saudi students, further extending its inquiry to assess the efficacy of Guidance and Counseling Programs in mitigating these issues. The findings contribute significantly to the growing body of literature addressing the psychological implications of social media use, particularly within the context of the Saudi Arabian academic setting. This research elucidates the interconnections of FoMO and SMA among Saudi students and underscores the effectiveness of Guidance and Counseling Programs in ameliorating these issues. The study emphasizes the importance of holistic approaches to student well-being, combining educational and psychological interventions. As the digital landscape continues to evolve, fostering a healthy relationship between students and social media requires ongoing attention and proactive measures from educational institutions and mental health professionals.

## Informed consent

Informed consent was obtained from all individual participants included in the study.

## Availability of data

The author confirms that data is available when it needed. To get the data that used in this research, please contact the corresponding author through this email: alabyd62@gmail.com.

## Ethical statement

Ethical considerations for this study on strategies and meditation programs encompass the assurance of participant confidentiality, voluntary participation, informed consent, and adherence to established research ethics guidelines. we get permission from the ethics committee of the Prince Sattam Bin Abdulaziz University, Saudi Arabia.

## Funding

The author extends his appreciation to 10.13039/100009392Prince Sattam bin Abdulaziz University for funding this research work through project number (10.13039/100009392PSAU/2023/02/25309).

## Declaration of competing interest

The authors declare the following financial interests/personal relationships which may be considered as potential competing interests:Mohammed Hasan Ali Al-Abyadh reports financial support was provided by 10.13039/100009392Prince Sattam bin Abdulaziz University. Mohammed Hasan Ali Al-Abyadh reports a relationship with Prince Sattam bin Abdulaziz University that includes: employment. The authors declare that they have no conflict of interest If there are other authors, they declare that they have no known competing financial interests or personal relationships that could have appeared to influence the work reported in this paper.
